# Challenges in the management of intracranial hypertension in pediatric patients with HIV: a case report

**DOI:** 10.3389/fmed.2025.1686021

**Published:** 2025-11-03

**Authors:** Amelia Cifuentes, Karolina Arellano, Nathaly Lapo, Jorge Vásconez-Gonázlez, Juan S. Izquierdo-Condoy, Esteban Ortiz-Prado

**Affiliations:** ^1^One Health Research Group, Universidad de las Américas, Quito, Ecuador; ^2^Hospital Baca Ortiz, Quito, Ecuador

**Keywords:** cryptococcosis, pediatric HIV, intracranial hypertension, serial lumbar puncture, central nervous system

## Abstract

**Introduction:**

The HIV epidemic is often primarily associated with key populations at higher risk of transmission, which has led to an underestimation of its impact on the pediatric population. In children, the disease may go unnoticed due to the delayed onset of symptoms, and diagnosis is often made at advanced stages, once opportunistic infections have already appeared such as cryptococcal meningitis, which carries high morbidity and mortality rates.

**Case report:**

A 13-year-old adolescent, newly diagnosed with WHO clinical stage IV HIV infection (unknown transmission route), was admitted with a central nervous system opportunistic infection caused by *Cryptococcus neoformans*. The patient presented with fever and seizures; the diagnosis was confirmed by positive India ink staining. Management, led by the Pediatric Infectious Diseases and Neurology services, focused on controlling intracranial hypertension (ICH) through 12 successive lumbar punctures for cerebrospinal fluid (CSF) drainage. Despite this aggressive multidisciplinary approach, the patient developed bilateral blindness secondary to chronic papilledema. This case highlights the challenges of advanced HIV in adolescence and the crucial need for effective ICH management in cryptococcal meningitis.

**Discussion:**

Cryptococcosis is one of the most severe opportunistic infections in immunocompromised patients, and its management in children and adolescents lacks standardized guidelines, which further complicates treatment. In this case, the absence of an external CSF drainage system and the need for multiple lumbar punctures highlight the therapeutic limitations in managing intracranial hypertension in resource-limited settings. This patient’s history shows that with appropriate care and a committed interdisciplinary team, it is possible to improve the quality of life for pediatric HIV patients, even in complex clinical scenarios.

**Conclusion:**

Early detection of HIV in children and adolescents must be strengthened, along with optimizing access to timely treatment to reduce the impact of opportunistic infections. Ongoing training should be promoted for healthcare professionals managing immunodeficient patients, as adherence to updated protocols and an interdisciplinary approach can make a significant difference in the prognosis and quality of life of these patients.

## 1 Introduction

The human immunodeficiency virus (HIV) belongs to the *Lentivirus* genus within the *Retroviridae* family and is classified into types 1 and 2 (HIV-1, HIV-2) (1). Globally, it is estimated that around 38 million people were living with HIV, of whom 1.8 million were children under the age of 15. In 2019 alone, of the 1.7 million new infections, 9% occurred in children (2). Ninety percent of children with HIV are infected through vertical transmission. The overall risk of vertical transmission in the absence of any intervention ranges from 20 to 45%. It is estimated that 5%–10% of transmissions occur *in utero*, 10%–20% during labor and delivery (intrapartum), and 5%–20% through breastfeeding (3). Without any intervention, the risk of HIV transmission from an infected mother to her child ranges from 15 to 45%. However, there are currently several strategies available to prevent mother-to-child transmission of HIV. This preventive approach is multifaceted and includes routine HIV testing for pregnant women. The Centers for Disease Control and Prevention (CDC) recommend HIV testing during every pregnancy, ideally at the first prenatal visit. Additionally, HIV testing is advised in the third trimester for certain high-risk situations, and at the time of delivery if testing has not yet been performed (4–6). For HIV-positive mothers, the administration of antiretroviral therapy (ART) is essential to reduce viral load. In cases where the viral load exceeds 1,000 copies/mL during pregnancy, cesarean delivery is recommended to decrease the risk of perinatal transmission (4, 5). Furthermore, after birth, infants born to HIV-positive mothers should receive antiretroviral prophylaxis. Those at higher risk of perinatal HIV transmission should continue treatment for up to 6 months (7). It is also necessary to perform diagnostic testing for HIV exposure at 14–21 days of life, again at 1–2 months, and subsequently at 4 and 6 months of age (7).

Cryptococcal meningitis (CM) is an opportunistic fungal disease caused by pathogenic yeasts of the *Cryptococcus* genus, with *C. neoformans* being the most frequently isolated species. It is a rare cause of meningitis in children (8, 9). A study conducted in Brazil involving 170 children with HIV identified 4 cases of CM. Similarly, Nyazika reported 4 cases over a 1-year period in sub-Saharan Africa (8, 9). It is estimated that around 75% of cases develop intracranial hypertension, due to the accumulation of capsular polysaccharide deposits and encapsulated yeasts in the arachnoid villi, perivascular spaces, and cerebral parenchyma, (10), however, these data primarily come from studies conducted in adult populations. Regarding the pediatric population, the available information is limited to a single case report published in 1996, which describes a case of intracranial hypertension and cryptococcal meningitis in a young girl with AIDS (11).

## 2 Case report

We present a case of newly diagnosed HIV infection in an adolescent: a 13-year-old male who presented 2 months prior to hospital admission with diffuse holocranial headache, rated 10/10 on the Visual Analogue Scale (VAS), accompanied by fever, generalized diaphoresis, and generalized tonic–clonic seizures. He was initially evaluated at a local hospital, where HIV testing was performed as part of the diagnostic workup due to the presence of meningeal signs in an adolescent with no known risk factors. The result was positive, representing the first time the patient received this diagnosis.

The exact timing and mode of HIV acquisition remain unknown, as the patient denied any history of needle sharing or intravenous drug use, and no history of sexual assault has been reported. There are no known cases of HIV infection within the patient’s family. In addition, the patient reports not being sexually active. Given the suspicion of a neuroinfection, the patient was subsequently referred to a tertiary care center for specialized management. It is important to note that, as neither the patient nor his legal guardians were previously aware of the HIV infection, the patient was not receiving any form of antiretroviral therapy at the time of admission (Viral load: 4050).

**FIGURE 1:**
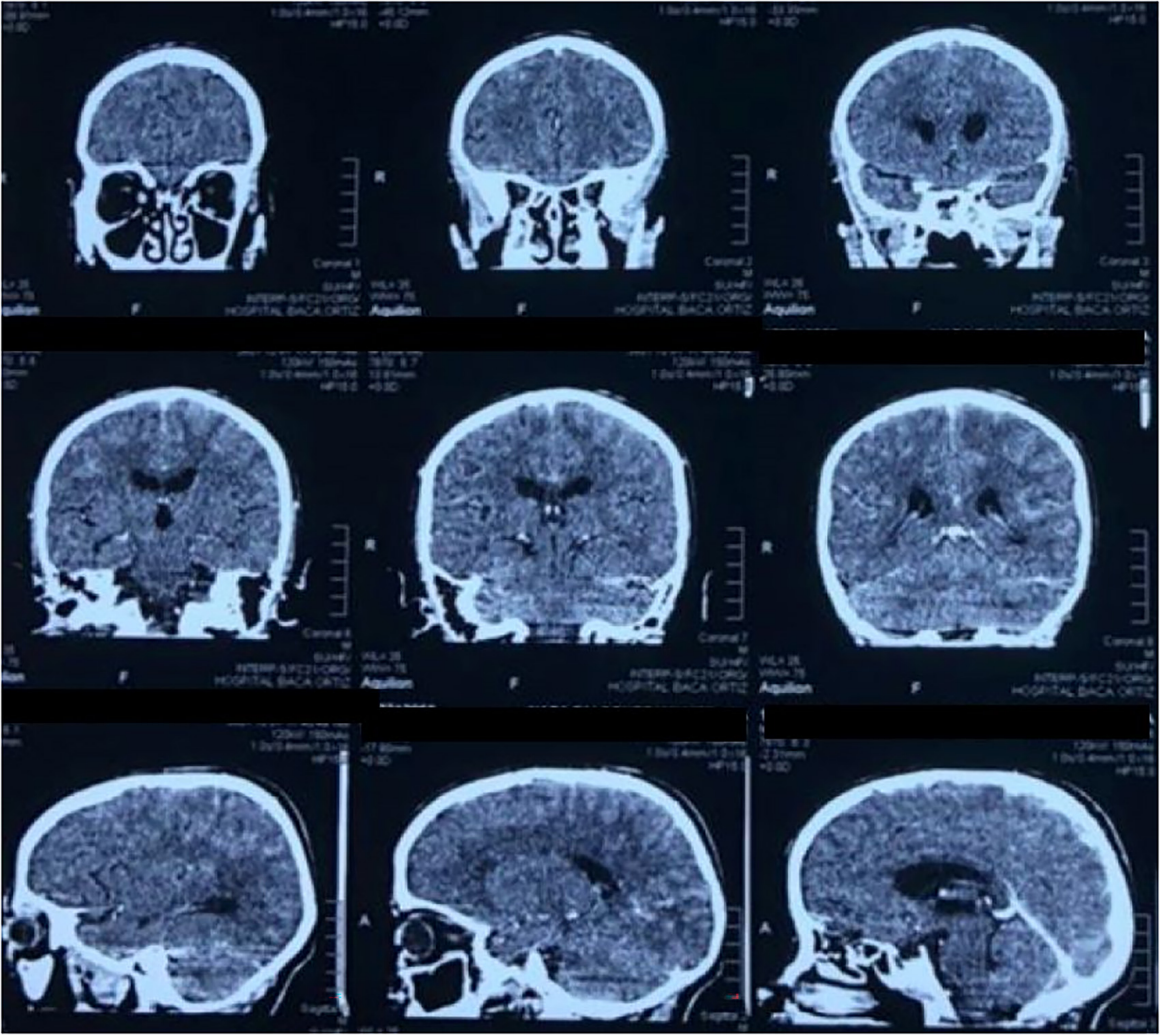
CT scan of the brain in coronal and sagittal planes shows a ventricular system with preserved morphology. No meningeal enhancement is observed.

Neurological examination revealed nuchal rigidity and positive Kernig and Brudzinski signs. Laboratory tests showed an HIV viral load of 4,050 copies/mL and a CD4 count of 11 cells/mm^3^, consistent with WHO clinical stage IV AIDS. Cerebrospinal fluid (CSF) analysis revealed hypoglycorrhachia, positive India ink staining, and cultures positive for *Cryptococcus neoformans* in both CSF and blood. A FilmArray panel also confirmed the presence of *Cryptococcus neoformans*. A cranial computed tomography (CT) scan performed at admission showed no ventricular abnormalities or meningeal enhancement (Figure 1).

The patient received induction therapy with intravenous amphotericin B, followed by consolidation and maintenance therapy with oral fluconazole. Antiretroviral therapy (TLD: tenofovir, lamivudine, and dolutegravir) was initiated 15 days after hospital admission. After slight clinical improvement, he was discharged following a 2-month hospitalization. Tuberculosis screening with QuantiFERON returned an indeterminate result, and testing for other opportunistic infections was negative.

One month after discharge, the patient was readmitted due to a clinical episode of herpes zoster and was treated with intravenous acyclovir, after which he was discharged again.

**FIGURE 2:**
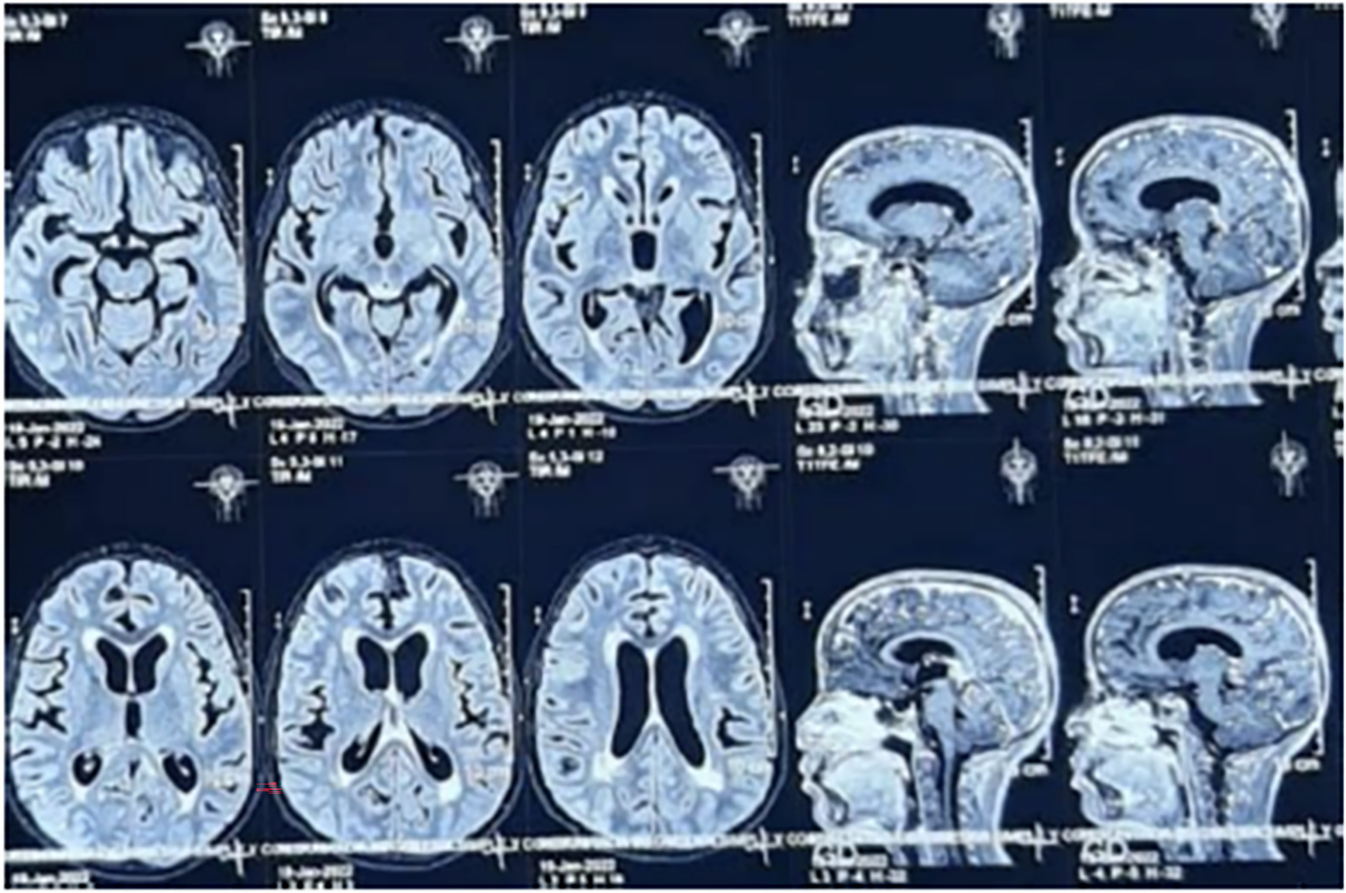
MRI without contrast shows evidence on T2-weighted sequences of abnormal contrast enhancement of the cerebral and cerebellar leptomeninges, cortical and central cerebral atrophy, and ventricular system dilation.

In the following weeks at home, his clinical condition deteriorated, with anxiety episodes, worsening and altered seizure patterns, recurrent fever, more intense holocranial headaches, and photophobia. He was readmitted for hospitalization; brain magnetic resonance imaging (MRI) during this admission revealed ventricular dilation and patchy hyperintensities in the periventricular white matter, without signs of cerebrospinal fluid flow obstruction findings consistent with intracranial hypertension (Figure 2).

Due to the presence of an active infection and the unclear etiology of the hydrocephalus, external ventricular drainage was not performed. The patient’s symptoms persisted and progressed to neuropsychiatric manifestations, including hallucinations, psychosis, and psychomotor agitation. The Neurology Department initiated therapeutic lumbar punctures twice weekly, later reduced to once weekly, for a total of 12 procedures. Initially, elevated opening pressures were recorded, followed by a progressive decrease.

Follow-up brain MRI showed progressive cerebral atrophy, stable ventricular dilation, and sequelae of the previous infectious process, with no worsening of the hydrocephalus. The clinical picture was classified as a relapse due to the persistence of febrile inflammatory symptoms and radiological signs of intracranial hypertension. This was interpreted as a manifestation of immune reconstitution inflammatory syndrome (IRIS). By the end of the second month of the second cycle of antifungal therapy, India ink staining became negative.

During his final hospitalization, which lasted 4 months, cytomegalovirus (CMV) coinfection was also identified and treated with valganciclovir. He developed healthcare-associated infections, with blood cultures positive for *Pseudomonas aeruginosa* and later *Acinetobacter baumannii*, both managed with targeted antibiotic therapy with favorable clinical response.

As an outpatient, a follow-up QuantiFERON test returned positive, prompting initiation of tuberculosis treatment with rifapentine and isoniazid.

In the clinical context of immune recovery following the initiation of antiretroviral therapy, the patient developed a paradoxical or unmasking form of IRIS, characterized by a sustained cerebral inflammatory response. It is noteworthy that ventricular dilation had not been observed on the cranial CT scan prior to ART initiation. Cerebrospinal fluid interleukin-6 levels were elevated (61 pg/mL), and the patient showed clinical improvement following the introduction of systemic corticosteroids.

Currently, the patient presents with bilateral blindness, classified as 80% visual disability, secondary to chronic papilledema. He remains under regular outpatient follow-up and continues antiretroviral therapy with good adherence.

## 3 Discussion

This case presents a pediatric patient diagnosed with HIV infection and cryptococcal meningitis, a severe opportunistic infection that, in this instance, manifested with an overwhelming cerebral inflammatory response. This response was triggered by the severity of the infection and the immune reconstitution following the initiation of antiretroviral therapy, resulting in intracranial hypertension and optic nerve involvement. Late diagnosis of HIV in childhood and adolescence remains a challenge, as the disease often presents with non-specific symptoms, delaying both its identification and the timely initiation of appropriate treatment.

World Health Organization (WHO) proposes guiding principles for the management of cryptococcal meningitis in adults, adolescents, and children living with HIV. For patients with persistent or recurrent symptoms, it recommends a thorough review of the medical history to identify potential causes of treatment failure. This includes assessing the dosage and duration of the pharmacological regimen, evaluating non-adherence or poor compliance during the fluconazole consolidation phase, and considering possible fluconazole resistance (12). Furthermore, it recommends measuring the opening pressure during lumbar puncture to detect intracranial hypertension and conducting microbiological evaluation of the cerebrospinal fluid to assess for co-infection or persistent infection (13, 14). It is important always considering the possibility of Immune Reconstitution Inflammatory Syndrome (IRIS) (15).

Given the presentation of intracranial hypertension in a pediatric patient with communicating hydrocephalus and no clear obstructive focus, and considering the severe immunosuppression associated with the HIV diagnosis, the decision was made to prioritize risk minimization and systemic stability over invasive surgical intervention. Therefore, serial lumbar punctures were preferred (instead of placing an external ventricular drain or a ventriculoperitoneal shunt). A shunt would only be placed in the event of acute or refractory hydrocephalus unresponsive to less invasive measures, in order to avoid the significant risk of bacterial superinfection or other complications related to the patient’s condition. Additionally, Immune Reconstitution Inflammatory Syndrome (IRIS) was clinically and biochemically confirmed, and steroid treatment proved effective.

Regarding pharmacological management, the reviewed evidence recommends an optimal antifungal treatment strategy in three phases: a 15 day induction therapy, followed by an 8-week consolidation phase, and then a maintenance or suppressive phase lasting at least 1 year, to reduce the risk of relapse (16). The WHO guidelines recommended 7 days of amphotericin B deoxycholate and flucytosine-based therapy (followed by 7 days of fluconazole 1200 mg) as the preferred antifungal regimen for the induction phase of treatment of HIV-associated cryptococcal meningitis (12), which has been shown to improve survival compared to Amphotericin B alone. However, at the time of treatment initiation for the patient, Flucytosine was not available; therefore, based on available evidence, an antifungal regimen of Amphotericin B plus Fluconazole was administered.

In AIDS-associated cryptococcosis, increased intracranial pressure (ICP) is primarily caused by the obstruction of CSF drainage at the arachnoid villi and granulations due to large accumulations of cryptococcal cells, released polysaccharides, inflammation, or a combination of these factors. Several studies suggest a possible link between elevated ICP and short-term mortality following cryptococcosis; however, prior estimates of the impact of ICP management through therapeutic lumbar punctures on mortality are lacking (17). The outcomes observed in this patient, based on a direct evaluation of survival benefit from therapeutic lumbar punctures, strongly support current treatment guidelines that emphasize the importance of ICP management in cryptococcal meningitis (17).

## 4 Challenges in case management

The therapeutic approach to this patient encountered several limitations, particularly in the management of intracranial hypertension:

-Limited availability of medical supplies: The absence of devices for monitoring cerebrospinal fluid (CSF) opening pressure significantly hindered proper assessment and follow-up.

-Lack of external CSF drainage: According to international guidelines, intracranial pressure in patients with cryptococcal meningoencephalitis should be managed aggressively to reduce mortality. However, external CSF diversion was not performed in this case, resulting in the need for multiple lumbar punctures and suboptimal management of intracranial hypertension. This contributed to a severe complication: optic nerve damage due to chronic papilledema and probable optic nerve ischemia, leading to 80% visual loss.

## 5 Impact and reflections on quality of care

Despite the challenges faced during his treatment, the patient was able to make a meaningful recovery, thanks to the unwavering support of his family and the Comprehensive Care Unit for People Living with HIV. Today, he has graduated from high school in his hometown, actively participates in his community by playing the piano at his church, goes to the gym with friends, is fully aware of his diagnosis, and maintains good adherence to his treatment regimen.

It is essential to acknowledge the dedication of the medical team, who, despite the complexity of the case, sought expert guidance and acted with professionalism, ethical responsibility, and strong commitment.

## 6 Conclusion

The delay in the management of intracranial hypertension resulted in severe visual impairment (80%), which might have been prevented with earlier intervention and access to appropriate cerebrospinal fluid (CSF) drainage devices. Due to a variety of social and environmental factors, patients with innate or acquired immunodeficiencies are at increased risk of exposure to multiple infectious agents. In this case, cryptococcosis was likely acquired through environmental exposure to birds and fruit, and latent tuberculosis was suspected, although the index case of exposure remains unidentified.

The source of HIV infection remains unknown, which contributed to the progression to AIDS before diagnosis. All family members were tested for HIV, with negative results. The patient achieved a positive outcome thanks to a well-structured and supportive family unit, the commitment and efficiency of the Comprehensive HIV Care Unit, and consistent interdisciplinary, patient-centered follow-up. His story underscores the importance of continuing efforts to strengthen education and awareness among healthcare professionals regarding the management of HIV in children and adolescents, ensuring that clinical decisions are guided by the best available evidence.

## Data Availability

The original contributions presented in this study are included in this article/supplementary material, further inquiries can be directed to the corresponding author.

## References

[1] German Advisory Committee Blood (Arbeitskreis Blut), Subgroup ‘Assessment of Pathogens Transmissible by Blood’. Human immunodeficiency virus (HIV). *Transfus Med Hemother.* (2016) 43:203–22. 10.1159/000445852 27403093 PMC4924471

[2] Nalwanga D Musiime V. Children living with HIV: a narrative review of recent advances in pediatric HIV research and their implications for clinical practice. *Ther Adv Infect Dis.* (2022) 9:20499361221077544. 10.1177/20499361221077544 35186289 PMC8855388

[3] World Health Organization. *Introduction: Infants, Children and HIV-Infection. In: Manual on Paediatric HIV Care and Treatment for District Hospitals: Addendum to the Pocket Book of Hospital Care of Children.* Geneva: World Health Organization (2011). 26131545

[4] NIH. *Prevención de la Transmisión Perinatal del VIH | NIH [Preventing Perinatal HIV Transmission | NIH].* Bethesda, MA: NIH (2025). Spanish.

[5] Siberry G. Preventing and managing HIV infection in infants, children, and adolescents in the United States. *Pediatr Rev.* (2014) 35:268–86. 10.1542/pir.35-7-268 24986927 PMC4071508

[6] World Health Organization. Prevention of HIV infection in children. In: Muhe L Gayer M Moss W editors. *Manual for the Health Care of Children in Humanitarian Emergencies.* Geneva: World Health Organization (2008).

[7] NIH. *Prevención de la Transmisión Perinatal del VIH Después del Parto | NIH [Preventing Perinatal HIV Transmission After Birth | NIH].* Bethesda, MA: NIH (2024). Spanish.

[8] Kaur R Rawat D Kakkar M Monga R Sharma V. Cryptococcal meningitis in pediatric AIDS. *J Trop Pediatr.* (2003) 49:124–5. 10.1093/tropej/49.2.124 12729297

[9] Nyazika T Masanganise F Hagen F Bwakura-Dangarembizi M Ticklay I Robertson V. Cryptococcal meningitis presenting as a complication in HIV-infected children: a case series from Sub-Saharan Africa. *Pediatr Infect Dis J.* (2016) 35:979–80. 10.1097/INF.0000000000001212 27187754 PMC4987191

[10] Alanazi A Adil M Lin X Chastain D Henao-Martínez A Franco-Paredes C Elevated intracranial pressure in cryptococcal meningoencephalitis: examining old, new, and promising drug therapies. *Pathogens.* (2022) 11:783. 10.3390/pathogens11070783 35890028 PMC9321092

[11] Laverda A Ruga E Pagliaro A Pinello M Giaquinto C. Intracranial hypertension and cryptococcal meningitis in a girl with AIDS. *Brain Dev.* (1996) 18:330–1. 10.1016/0387-7604(96)00022-8 8879656

[12] World Health Organization. *Guidelines for Diagnosing, Preventing and Managing Cryptococcal Disease Among Adults, Adolescents and Children Living with HIV.* Geneva: World Health Organization (2022). 35797432

[13] Bicanic T Brouwer A Meintjes G Rebe K Limmathurotsakul D Chierakul W Relationship of cerebrospinal fluid pressure, fungal burden and outcome in patients with cryptococcal meningitis undergoing serial lumbar punctures. *AIDS.* (2009) 23:701–6. 10.1097/QAD.0b013e32832605fe 19279443

[14] Graybill J Sobel J Saag M van Der Horst C Powderly W Cloud G Diagnosis and management of increased intracranial pressure in patients with AIDS and cryptococcal meningitis. The NIAID mycoses study group and AIDS cooperative treatment groups. *Clin Infect Dis.* (2000) 30:47–54. 10.1086/313603 10619732

[15] Schregenberger S Graup V Schibli A Preiswerk B Laube I Huber L Immune reconstitution inflammatory syndrome (IRIS): case series and review of the literature. *Respir Med Case Rep.* (2025) 55:102213. 10.1016/j.rmcr.2025.102213 40276120 PMC12019413

[16] Chang C Harrison T Bicanic T Chayakulkeeree M Sorrell T Warris A Global guideline for the diagnosis and management of cryptococcosis: an initiative of the ECMM and ISHAM in cooperation with the ASM. *Lancet Infect Dis.* (2024) 24:e495–512. 10.1016/S1473-3099(23)00731-4 38346436 PMC11526416

[17] Rolfes M Hullsiek K Rhein J Nabeta H Taseera K Schutz C The effect of therapeutic lumbar punctures on acute mortality from cryptococcal meningitis. *Clin Infect Dis.* (2014) 59:1607–14. 10.1093/cid/ciu596 25057102 PMC4441057

